# Traumatic quadriceps rupture in a patient with patellectomy: a case report

**DOI:** 10.1186/1752-1947-1-146

**Published:** 2007-11-24

**Authors:** Chezhiyan Shanmugam, Nicola Maffulli

**Affiliations:** 1 Department of Trauma & Orthopaedic Surgery, University Hospital of North Staffordshire, Keele University School of Medicine, Stoke-on-Trent, ST4 6QG, UK

## Abstract

**Introduction:**

Acute traumatic, unilateral, quadriceps rupture after patellectomy is rare.

**Case presentation:**

We present a 42-year old male who experienced a unilateral left quadriceps tendon rupture following assault by four people. Twenty-seven years before this injury, the patient had suffered ipsilateral femur and comminuted patellar fractures, which were managed by intramedullary nailing and patellectomy respectively. We performed primary end to end repair of the torn tendon. Postoperatively, histology revealed findings consistent with pre-existent degenerative changes. The patient made good recovery, and returned to his former occupation which was reliant on his ability to drive.

**Conclusion:**

Degenerative changes of the tendon of the extensor mechanism of knee following patellectomy may predispose the quadriceps tendon to traumatic rupture. Early operative intervention and protracted rehabilitation are required to obtain the best functional results.

## Introduction

Unilateral post-traumatic quadriceps tendon rupture after patellectomy is rare. Quadriceps tendon rupture is more commonly seen in patients older than 40. The tendon usually fails above the osteo-tendinous junction in 70% of patients, and in the intra-tendinous substance in the remaining 30%. A strong association exists with numerous systemic diseases and prior degenerative changes in the knee extensor mechanism [1]. Reconstruction of a chronic patellar tendon rupture after patellectomy has been reported [2].

## Case presentation

**Figure 1:**
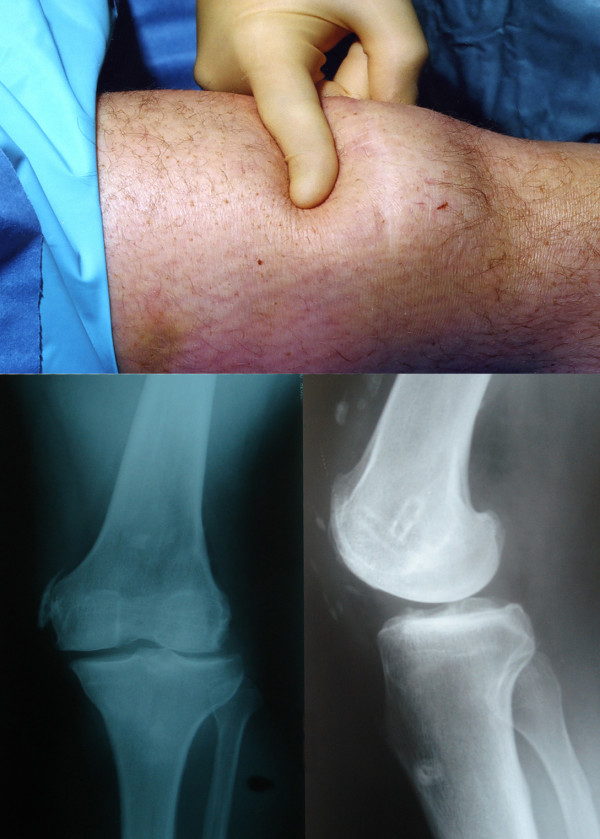
Clinical picture showing positive "sulcus sign" in the suprapatellar region, and the radiographs showing heterotopic ossification, absence of patella, and soft tissue shadow defect anterior to the distal femur.

A 42-year-old male was assaulted by four persons, and remembers that some of them jumped on his left knee. He was not sure about the exact mechanism of injury, as he stated that he was under the influence of alcohol. The patient presented with swelling of his thigh and inability to weight bear on the left leg. There was bruising and a palpable depression (Fig 1) of the left quadriceps tendon. The patient was able to extend the knee, but not against gravity, with an extensor lag of 30°.

The patient reported that, 27 years before the present injury, he had been involved in a road traffic accident sustaining ipsilateral femur and comminuted patellar fractures. The femur underwent intramedullary nailing. The patella was removed, and primary repair of the extensor mechanism was performed. Six years later, the intramedullary femoral nail was removed. In the patient's past medical history, there were no other significant problems to note, and there was no history of quinolones intake. The patient had been generally fit and well.

Plain radiographs of the knee showed a depression in the soft tissue shadow anterior to the distal femur, extraosseus calcification along the tendon, and absence of the patella (Fig 1). Other imaging investigations were not performed, as the clinical signs were classical.

**Figure 2:**
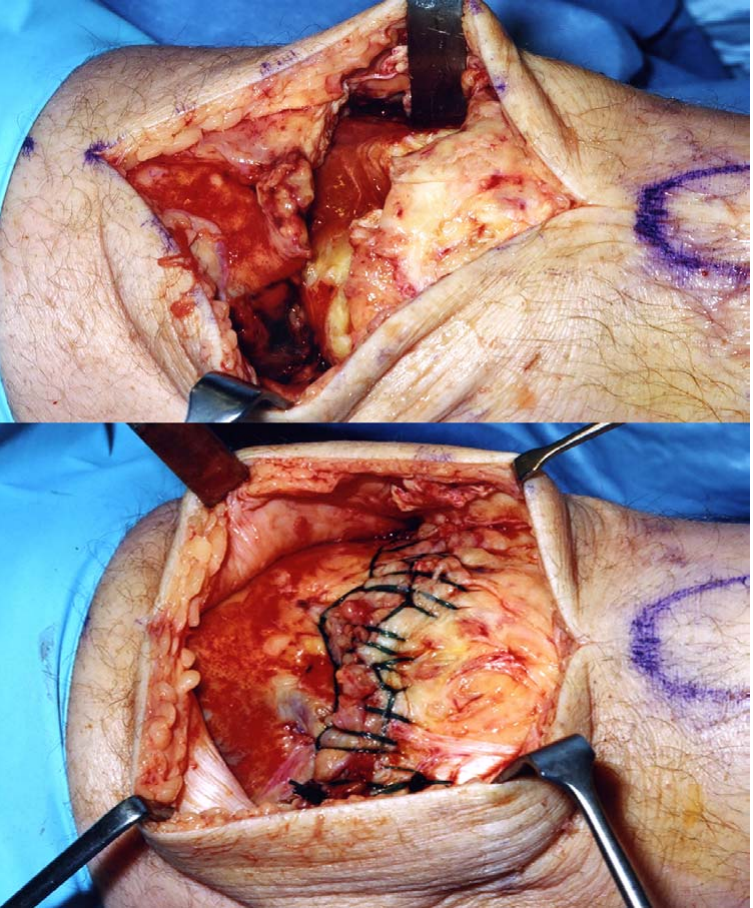
Complete quadriceps tear and the end to end primary repair using continuous locked sutures.

**Figure 3:**
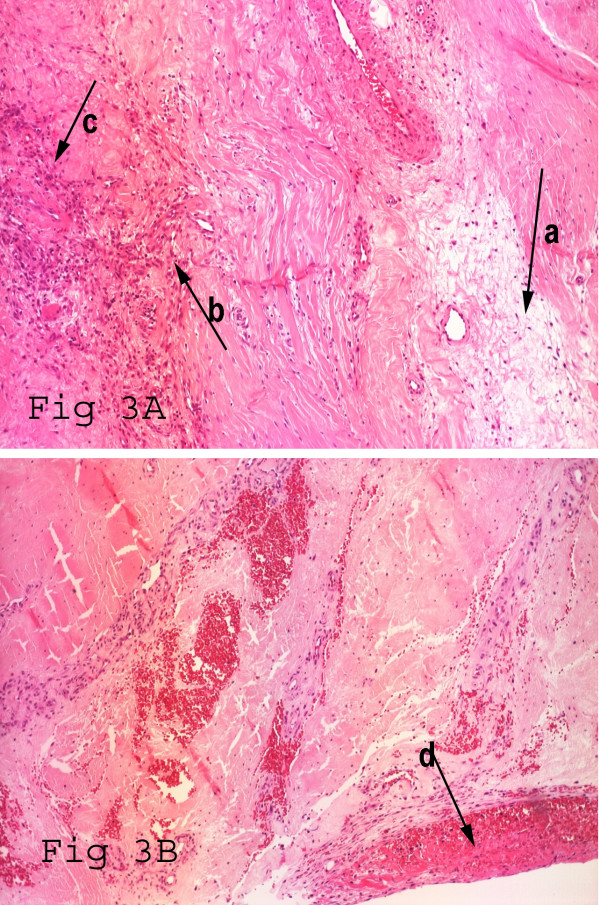
**(Hematoxylin and Eosin stain × 200 Magnification)**. A. Histology showing features of hypoxic degenerative tendinopathy at the rupture site. B. Contiguous microscopic section of adjacent area depicted in part A, showing fibrin at the rupture site. Slide labels: a. Disorganisation of collagen fibrils and increased ground substance (mucin like material). b. Lymphocyte infiltration. c. Neovascularisation. d. Fibrins at the rupture site.

We elected to perform a primary quadriceps repair within 24 hours of the injury. A midline incision was made over the previous scar. A complete quadriceps rupture was noted approximately 3 cm proximal to the patellectomy site with a 2.5 cm gap (Fig 2). The previous extensor mechanism repair site was well healed. The medial and lateral retinaculums were intact. An end to end repair was performed using continuous, locked heavy absorbable sutures (size 1 Maxon-Monofilament polygyconate synthetic suture, United States Surgical, USA) in multiple layers (Fig 2). The ragged margins of the tendon were excised and sent for histopathological examination. This showed disorganisation of collagen fibrils and increased ground substance (mucin like material), lymphocyte infiltration, neovascularisation and fibrins at the rupture site. These findings were consistent with chronic hypoxic tendon degeneration following patellectomy (Fig 3A), on which superadded changes from the rupture were found (Fig 3B).

The repair was protected with a knee brace for 6 weeks. During that period, the patient was allowed partial weight bearing mobilisation with one crutch during the initial 6 weeks, and advised to wean it off gradually over a period of 2–3 weeks time [3]. He was encouraged to put full weight bearing at the time of weaning the crutch. The knee brace was removed after 12 weeks. We advised him to refrain from work for 12 weeks. Post-operative recovery was complicated by a superficial infection which settled with 2 weeks of oral antibiotics. We encouraged the patient to start straight leg raise exercises with the knee brace, and gravity assisted passive knee bending from the 4^th ^week. Physiotherapist started closed chain exercises at 8 weeks; and open chain exercises at 12 weeks, and were increased in intensity and duration at 24 weeks of surgery.

At 7 months from the injury, there was no extension lag, with a range of active knee flexion from 0° to 100°. Further flexion was restricted by tightness, mild discomfort and pain. The isometric extensor strength of the knee extensors was measured [4]. The mean of three measurements was taken, and compared to the normal side. The knee extensor strength was (771 N) 44% less when compared to contralateral normal quadriceps strength (1386 N).

The overall functional outcome was measured using the International Knee Documentation Committee (IKDC) knee score. This was 121.6 before injury and 51.8 after surgery. A score of 100 is interpreted to mean no limitation with activities of daily living or sports activities and the absence of symptoms. The patient is unable to play football, run and jog, but he is able to speed walk, and has returned to his pre-operative work (which was reliant on his ability to drive) 6 months after this injury.

## Discussion

Quadriceps rupture is relatively rare, most often occur in males older than 40 years [1,3]. Quadriceps tendon ruptures are more common than patellar tendon ruptures, but are more likely to be misdiagnosed [1,5]. To our knowledge, this is the first report of acute traumatic quadriceps rupture following patellectomy. In 1998, Wascher et al. [2] reported a patient with chronic rupture of the patella tendon after patellectomy, which was managed by Achilles tendon allograft.

Quadriceps tendon ruptures are described as being traumatic or idiopathic, or are associated with various systemic diseases. Quadriceps tendon rupture has also been reported as a complication of various surgical procedures of the knee for example after proximal quadriceps release in total knee arthroplasty, lateral retinacular release, meniscectomy, and anterior cruciate ligament reconstruction with central-third patellar tendon graft.

It is difficult to ascertain a cause-effect relationship between patellectomy and subsequent quadriceps tendon rupture. In our patient, histopathological examination of the quadriceps tendon at the rupture site is consistent with chronic degenerative changes. Radiographically, signs of calcifying tendinopathy were evident. Kannus et al. [6] in 1991 published the histopathological changes in 891 various ruptured tendons, among which 82 were quadriceps and patellar tendons. The study results showed that 97% of the pathologic changes were degenerative. Among the quadriceps group, hypoxic degenerative tendinopathy was the most common type of lesion, ranging from 21 to 49% in the ruptured tendons and from 5 to 17% in the control tendons [6]. In our patient, both calcifying and hypoxic degenerative tendinopathy were present.

Degenerative changes following patellectomy are common. We do not know why the vast majority of patients with patellectomy, who would have developed degenerative changes, do not experience a rupture. Probably, trauma may be an incidental provocative factor for the already degenerated tendon to rupture. Various factors implicated in degeneration of tendons have been described, including increased age, disturbances in micro- and macro-vascular circulation, severe soft tissue injury at the time of original trauma, overuse injury (tendinopathy), and altered biomechanics due to patellectomy, either alone or in combination.

In experiments on rabbit tendons, normal tendons will not rupture unless it suffers considerable damage [7]. Rupture did not occur even when one-half of a tendon was severed without obstruction to the blood supply. In humans, the superficial layers of the quadriceps tendon are well vascularised. However, in the deep layer, there is an oval 30 by 15 mm avascular area which may degenerate [8]. Any disturbances to the vascularity of the deep layer of the quadriceps tendon might be a contributing factor for tendon degeneration.

Tendons are at the highest risk for rupture if tension is applied quickly and obliquely, and the highest forces are seen during eccentric muscle contraction [9]. Harkness demonstrated that approximately 30 kg/mm^2 ^of longitudinal stress have to be applied to the normal quadriceps tendon prior to failure. Therefore, tendon rupture usually occurs through a pathologic area of the tendon, which may explain why many ruptures occur after relatively trivial trauma [8].

Extensor weakness is common following patellectomy. One may ask whether the extensor weakness of knee is secondary to patellectomy, or to degeneration. Ackroyd et al. [10] reported that, of 81 patients who had patellectomy for degenerative osteoarthritis, 50% had weakness of extension of the knee, and only 53% of their patients had a good result at a mean of 6.5 years after operation. In a similar study [11] on the long term functional outcome of patellectomy in 69 patients, function had remained at the same high level despite the patellectomy.

In our patient, old trauma and patellectomy would have altered the biomechanics of the extensor mechanism, subjecting the quadriceps tendon to undue stresses. Changes leading to microtrauma would result in tendon fibril damage, accelerating the cascade of degenerative changes, possibly predisposing the tendon to fail when subjected to direct or indirect trauma.

Management of acute quadriceps rupture following patellectomy is the same as that of any traumatic quadriceps rupture. MRI is useful in differentiating complete from partial quadriceps tendon tears, but it is expensive, and, in our settings, less readily available than ultrasound. Caution should be exercised to restore a moment arm, and centralise the extensor mechanism. Early surgical (primary end to end) repair yields the best results for complete quadriceps tendon ruptures. Delay in surgical management will produce worse functional results [12]. All surgical methods can be expected to give comparable results, provided that surgery is undertaken within 1 week of the injury [12,3]. Most patients with repaired quadriceps tendon can expect a good range of motion and return to their previous occupation. In our patient, return to pre-injury lifestyle is expected to take much longer because of recurrent trauma and surgery.

Delaying passive knee flexion up to 4 weeks would prevent the risk of early inadvertent stretching and failure of the repair. This delay will also minimise the discomfort and pain. Closed chain exercises can be started at 8 weeks. Some of the closed chain exercises including seated leg presses-one-third knee bends on one leg, then both, stationary bicycling and rowing machine exercises, may be delayed upto 12 weeks depending on individual recovery of function. Open chain exercises started at 12 weeks, and were increased in intensity and duration at 24 weeks. Protracted rehabilitation for more than 6 to 8 months should be anticipated.

We are not aware of any study that details the effect of assault of four men on a tendon. The published evidence points towards tendinopathy being present before tendon rupture. Physiological and biomechanical studies show that a normal tendon can be subjected to 17 times body weight loads without rupturing [13]. Obviously, we cannot rule out direct mechanical failure of a normal tendinous structure when subjected to beatings by four men. However, given the present state of scientific knowledge and the histological appearance of the ruptured tendon in our patient, we favour the aetiopathogenetic hypothesis that less than optimal tendon tissue was subjected to abnormally high mechanical forces, and therefore failed.

## Conclusion

Traumatic quadriceps rupture after patellectomy is rare. Patellectomy may induce degenerative changes in the extensor mechanism, and these degenerative changes may predispose the quadriceps tendon to traumatic rupture. Early operative intervention and protracted rehabilitation are required.

## Abbreviations

IKDC = International Knee Documentation Committee; 

N = Newton; 

USA = United States of America.

## Competing interests

The author(s) declare that they have no competing interests.

## Authors' contributions

CS wrote the case report including performing the literature review. NM is an experienced Trauma & Orthopaedic Surgeon with an interest in the tendon problems. NM provided guidance for the literature search, the writing of the paper, and also proof read the paper. Both authors have read and approved the final manuscript.

## Consent

Written consent was obtained from the patient for publication of the study.
